# Down-Regulation of Claudin-18 Is Associated with the Proliferative and Invasive Potential of Gastric Cancer at the Invasive Front

**DOI:** 10.1371/journal.pone.0074757

**Published:** 2013-09-20

**Authors:** Tadayuki Oshima, Jing Shan, Takuya Okugawa, Xin Chen, Kazutoshi Hori, Toshihiko Tomita, Hirokazu Fukui, Jiro Watari, Hiroto Miwa

**Affiliations:** Division of Upper Gastroenterology, Department of Internal Medicine, Hyogo College of Medicine, Nishinomiya, Hyogo, Japan; Vanderbilt University Medical Center, United States of America

## Abstract

**Background:**

Claudins are known as tight junction proteins, and their expression pattern in gastric cancer is still controversial. The relationship between the expression patterns of tight junction proteins and tumor proliferation in early gastric cancer is still far from clear.

**Aims:**

To investigate the expression patterns of claudin-18 and Ki-67 in early gastric cancer at the invasive front and surrounding normal gastric mucosa and to investigate the biological function of claudin-18 in the proliferation and invasion of cancer cells.

**Methods:**

Seventy-five early gastric cancer lesions removed via endoscopic mucosal resection or endoscopic submucosal resection were evaluated. All gastric cancer lesions were diagnosed as differentiated adenocarcinoma using the Japanese Classification of Gastric Carcinoma. To assess epithelial proliferation, immunostaining with Ki-67 was performed, and the labeling index was calculated. To assess the expression of epithelial tight junction proteins, immunofluorescent staining of claudin-18 was performed. The immunoreactivity of claudin-18 was graded according to the number of stained cells. Correlation analysis was performed by Spearman’s rank correlation coefficient. Transfection of claudin-18 small interfering RNA (siRNA) was accomplished in MKN74, a claudin-18-positive gastric cancer cell line, to investigate the effect of claudin-18 on proliferation and invasion of cancer cells.

**Results:**

Claudin-18 was significantly down-regulated in gastric cancer compared to surrounding gastric normal mucosa or intestinal metaplasia. The Ki-67 labeling index of gastric cancer at the invasive front was inversely correlated with the claudin-18 level, but that at the mucosal lesion was not correlated. Claudin-18 knockdown significantly promoted the proliferation of MKN74 compared with control siRNA-transfected cells. MKN74 invasion increased significantly with claudin-18 siRNA transfection compared with control siRNA transfection.

**Conclusions:**

Down-regulation of claudin-18 is associated with the proliferative potential at the invasive front of gastric cancer, suggesting that it has a pivotal role in gastric cancer progression.

## Introduction

Gastric cancer (GC) is the fourth most commonly diagnosed malignancy and the second most common cause of cancer-related mortality worldwide [1], [2]. Intramucosal GC can be curatively treated by endoscopic treatment. Furthermore, the endoscopic submucosal dissection (ESD) technique has expanded the indications for endoscopic treatment and enabled en bloc resection of intramucosal large and slightly submucosal invasive early GCs [3]. An en bloc resection by ESD enables accurate diagnosis of cancer depth [4].

Tight junctions (TJs) are specialized membrane domains at the most apical region of polarized epithelial and endothelial cells [5]. Claudins are the major TJ proteins; they consist of at least 27 member proteins and are expressed in a tissue-specific manner [6], [7]. They regulate paracellular permeability and establish epithelial cell polarity by their fence function [6], [8]. A growing body of recent evidence suggests that claudins are key structural and functional components of TJ strands [6], [9], and they may play a crucial role in tumorigenesis and inflammation [10], [11].

The gastric epithelium exhibits a series of changes in claudin expression from normal mucosa to intestinal metaplasia and adenocarcinoma. Although GCs were recently classified by mucin-based expression [12], [13], a claudin-based GC classification has also been proposed [14]. We have also reported that down-regulation of claudin-3 was related to the proliferation of intramucosal GC [15]. Recently, claudin-18 knock-out mice were shown to have gastric mucosal atrophy and paracellular H^+^ ion leakage [16]. Claudin-18a2 was shown to be frequently down-regulated in GC of an intestinal phenotype, suggesting that claudin-18a2 would be a good marker for GC [17]. However, the biological function of claudin-18, which might be involved in cancer cell behavior, has never been examined. Furthermore, it is not clear which characteristic of GC cells at the invasive front is related to the rapid growth or aggressiveness of invasion.

In the present study, the expression level of claudin-18 in GC at the invasive front and surrounding gastric mucosa was examined for the first time, and the correlation between the claudin-18 level and the Ki-67 labeling index (LI) in GC was evaluated. The effect of claudin-18 on proliferation and invasiveness *in vitro* was also examined.

## Materials and Methods

### Patients

A consecutive series of 74 patients with early GC were studied (Table 1). GC lesions (n = 75) that had undergone curative EMR or ESD were evaluated. GCs with submucosal invasion (n = 31) were included in this study. Double cancers were detected only in one male patient. All of the GC were classified as differentiated adenocarcinoma using the Japanese Classification of Gastric Carcinoma [18]. Patient anonymity was preserved. This study was performed in accordance with the declaration of Helsinki and was approved by the Ethics committee/Institutional Review Board of Hyogo College of Medicine. The subject gave written informed consent.

**Table 1:** Clinicopathological features of gastric cancer patients.

	Total (T1a and T1b)	T1b
Number of lesions (number of patients)	75 (74)	31 (30)
Age, year (mean ± SD)	71.5±8.8	70.1±7.5
Sex (male/female)	51/23	24/6
Location (U/M/L)	15/37/23	8/17/6
Size, mm (mean ± SD)	17.0±10.8	23.3±11.0
Histological type (tub1/tub2/pap)	60/14/1	18/12/1

T1a; tumor confined to the mucosa, T1b; tumor confined to the submucosa,

U; upper third, M; middle third, L; lower third [18]

### Immunohistochemistry

Samples were fixed with 10% formalin solution and embedded in paraffin. Sections were cut to 3 µm thickness and were stained with hematoxylin and eosin (HE). To assess epithelial proliferation, samples were incubated with Ki-67 monoclonal antibody (Invitrogen, Carlsbad, CA, USA) followed by incubation with biotinylated horse-anti-mouse secondary antibody (Vector Laboratories, Burlingame, CA, USA). The streptavidin-biotin complex method (Vector Laboratories) was used to visualize the immunoreactivity. Nuclei were counterstained with hematoxylin.

### Immunofluorescent Staining of Junctional Proteins

Rabbit anti-claudin-18 polyclonal antibody (Invitrogen) and Cy3-conjugated goat anti-rabbit IgG (Jackson ImmunoResearch Laboratories, West Grove, PA, USA) secondary antibody were used to visualize immunoreactivity. The specificity of the reaction was tested by incubation with non-immune rabbit serum (Dako, Glostrup, Denmark).

### Scoring of Immunostaining of Claudin-18 and Ki-67

Immunoreactivity was evaluated by two independent observers (T.Ok., X.C.), and both were blind to the clinicopathological features and clinical outcomes associated with each sample. Non neoplastic gastric mucosa without intestinal metaplasia (non-IM) and with intestinal metaplasia (IM) and cancer regions were assessed. The immunoreactivity of claudin-18 was graded using the following criteria: 0, absence of membrane staining; 1+, fewer than 10% of tumor cells with complete membrane stain; 2+, 10% to 50% of tumor cells with complete membrane staining; 3+, more than 50% to 75% of tumor cells with complete, strong membrane staining; and 4+, more than 75% of cells with complete, very strong membrane staining. Five high-power fields were analyzed in each region of the cases and the mean score was calculated. All of the sections were scored twice to confirm the reproducibility of the results. The Ki-67 LI was calculated after counting 1000 cancer cells.

### Small Interfering RNA (siRNA) Knockdown Experiments

For siRNA silencing of human claudin-18, ON-TARGET plus SMARTpool and a non-specific control siRNA was purchased from Dharmacon, Inc. (Lafayette, CO, USA). The MKN74 cell line (Japanese Collection of Research Bioresources Cell Bank, Osaka, Japan), which is *CLDN18*-positive, was selected to study the effects of claudin-18 knockdown on the proliferation and invasion properties of GC cells. Cells were transfected with 25 nM siRNA using DharmaFECTTM3 (Dharmacon, Inc.). Control and control siRNA groups were treated with the transfection reagents without siRNA and the transfection reagents with non-specific control siRNA, respectively. Assays were performed 3 days after the MKN74 cells were transfected.

### Cell Proliferation Assay

Cell proliferation was determined by a colorimetric assay of cell viability based on the cleavage of the tetrazolium salt WST-1 by mitochondrial dehydrogenases (Takara Bio Inc., Otsu, Japan). The absorbance of the formazan dye formed, which correlates with the number of metabolically active cells in the culture, was measured at 450 nm 1 h after adding the reagent.

### Invasion Assay

MKN74 cell invasion was assayed in 24-well Biocoat Matrigel invasion chambers (8 µm; Becton Dickinson, Franklin Lakes, NJ, USA) according to the manufacturer’s protocol. The lower chambers were filled with RPMI1640 with 10% FBS, and cells (2×10^5^) were placed in the insert chambers, which contained RPMI1640 without FBS. After 24-h incubation, the noninvasive cells were removed with a cotton swab. The cells that have migrated through the membrane and stuck to the lower surface of the membrane were fixed with 10% formalin and stained with hematoxylin. For quantification, cells were counted under a microscope in five random high-power fields. Assays were performed in triplicate. The invasion ratio to control medium was calculated.

### Statistical Analysis

Statistical evaluation was performed using SPSS13.0 (SPSS, Chicago, IL, USA). Correlation analysis was performed by Spearman’s rank correlation coefficient (rs). Multiple comparisons were performed using the Kruskal-Wallis test followed by Scheffe’s test. Single comparisons were performed by the Mann-Whitney test. *P*<0.05 was considered significant.

## Results

### Expression of Claudin-18 in Gastric Mucosa and Carcinoma

**Figure 1:**
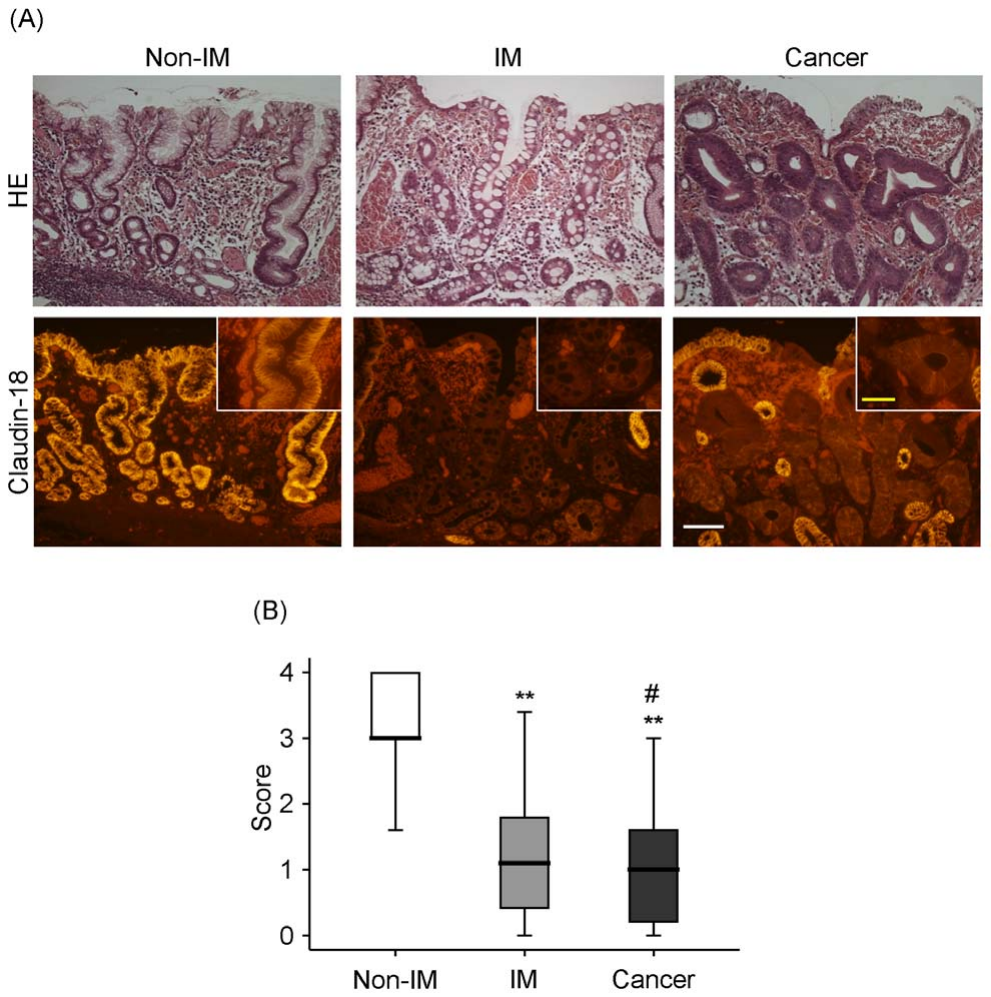
HE staining and immunofluorescence staining of claudin-18 in gastric cancer (GC) lesions and surrounding gastric mucosa. (A) HE staining of gastric mucosa with non-intestinal metaplasia (non-IM), intestinal metaplasia (IM), and cancer. Claudin-18 is detected in gastric mucosa with non-IM at the surface border and lateral membrane of the epithelial cells, but not with IM or cancer. White bar = 100 µm, Yellow bar = 50 µm. (B) Claudin-18 level is significantly lower in GC than in non-IM or IM. The level is also significantly lower in IM than in non-IM. ^#^ *P*<0.05 vs. IM. **P<0.01 vs. non-IM.

A total of 75 differentiated GC cases were analyzed in this study. The sections were stained with claudin-18, and the level of staining was graded. Claudin-18 was detected in high levels in gastric epithelial cells at the apical and lateral side borders in non-IM but not in IM or differentiated adenocarcinoma cells (Fig. 1A). The levels of claudin-18 were significantly lower in cancer cells than in non-IM and IM, as well as significantly lower in IM compared to non-IM (Fig. 1B).

### Detection of Claudin-18 in GCs with Submucosal Invasion

**Figure 2:**
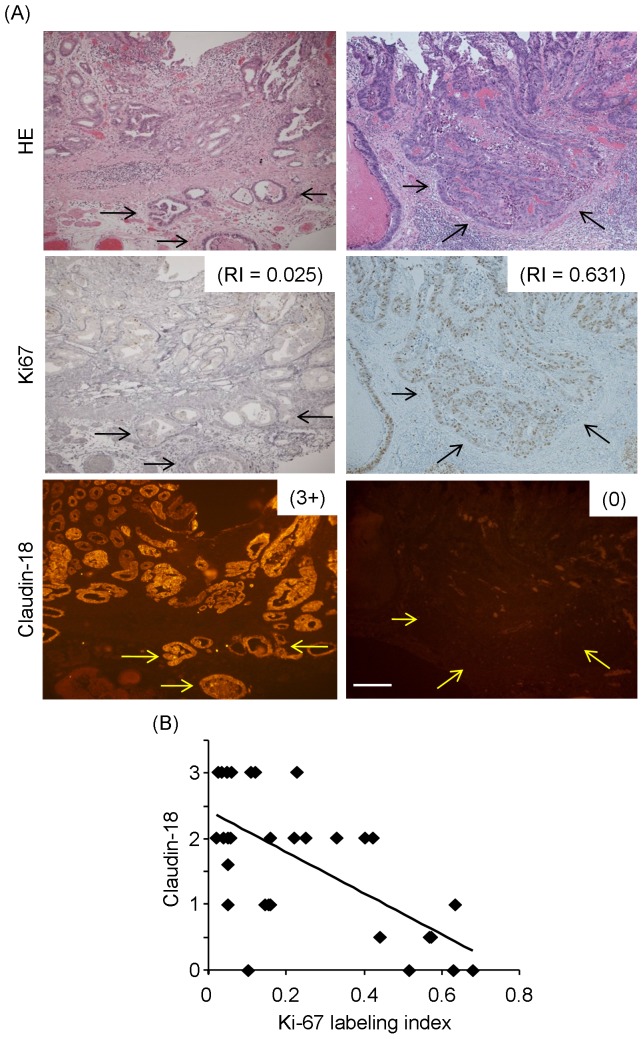
Detection of claudin-18 in mucosal and submucosal lesions of GC and the Ki-67 LI. HE staining shows cases of submucosal invasive GC. Ki-67 positive nuclei are detected in the cancer cells. The Ki-67 LI is low in a case of submucosal-positive claudin-18 staining (3+) (left) and high in a case of submucosal-negative claudin-18 staining (0) (right). Bar = 100 µm. (B) The Ki-67 LI is inversely correlated with the claudin-18 level (rs = −0.550, *P* = 0.0010).

Representative GC cases with submucosal invasion showed that dense claudin-18 staining was evident at the invasive front of submucosal invaded-cancer cells, and Ki-67 LI was low at the lesion (Fig. 2A). In a case with low claudin-18 staining at the invasive front of submucosal invaded-cancer cells, Ki-67 LI was high at the lesion (Fig. 2A).

### Correlation of the Claudin-18 Level and Ki-67 LI

The Ki-67 LI of GC in mucosa and submucosa was determined by the percentage of Ki-67-positive nuclei. To determine the correlation between the Ki-67 LI and the claudin-18 level, correlation analysis was performed. The mucosal expression level of claudin-18 was not significantly correlated with the Ki-67 LI (rs = 0.07, *P = *0.60). On the other hand, the submucosal claudin-18 expression level was significantly inversely correlated with the submucosal Ki-67 LI (rs = −0.550, *P* = 0.0010) (Fig. 2B).

### Effect of Claudin-18 siRNA on the Proliferation and Invasion of GC Cells

**Figure 3:**
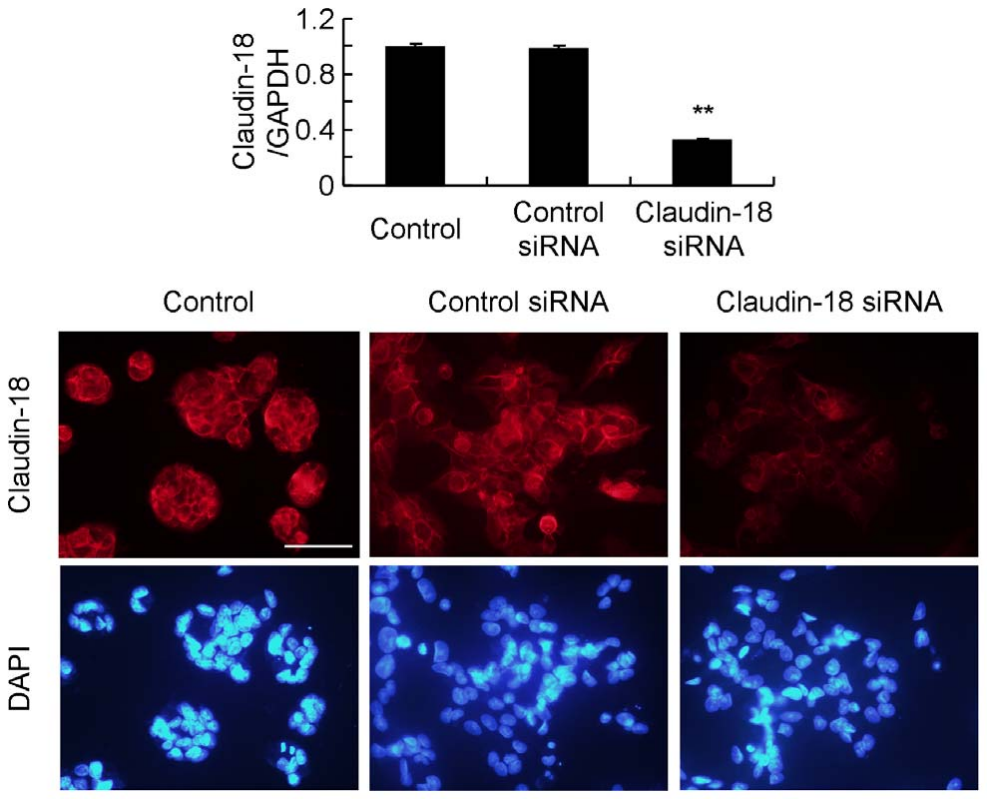
Claudin-18 knockdown by siRNA. (A) Claudin-18 siRNA significantly decreases the mRNA level of claudin-18 in MKN74. (B) Staining of claudin-18 is reduced by claudin-18 siRNA treatment. Bar = 50 µm. Each value represents the mean ± SD of 3 independent experiments. **P<0.01 vs. control or control siRNA.

**Figure 4:**
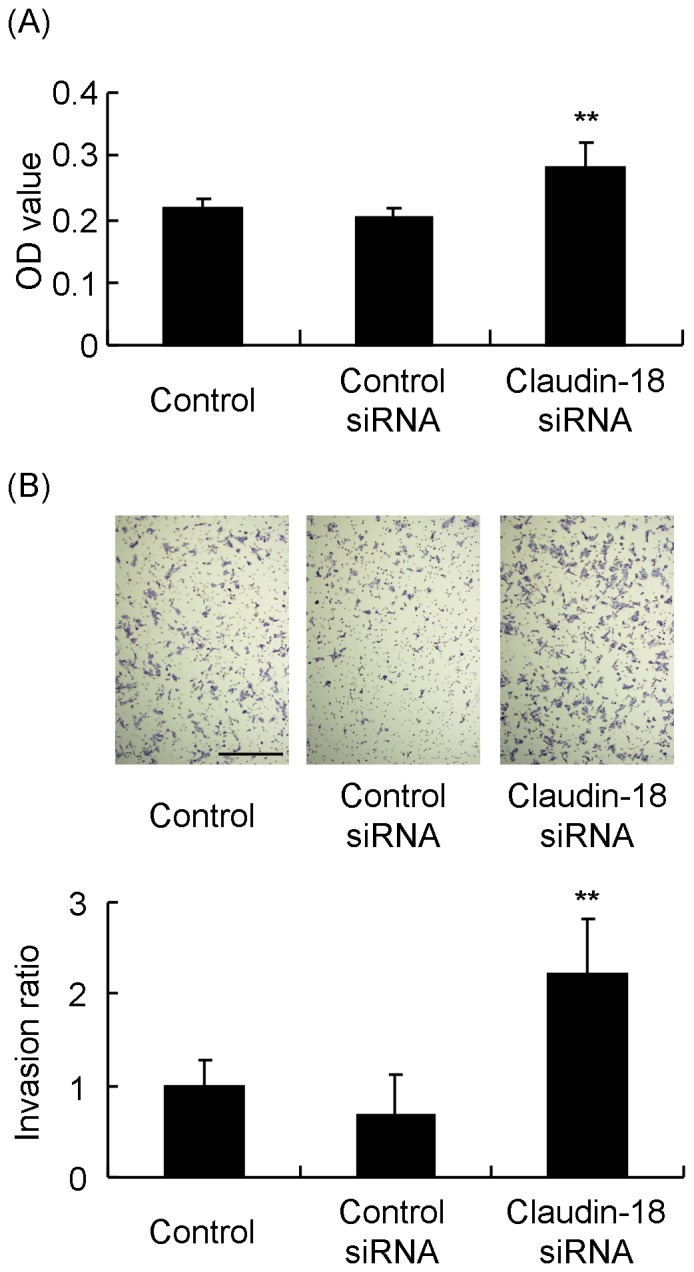
Effect of claudin-18 knockdown on the proliferation and invasion of GC cells. (A) Claudin-18 siRNA treatment significantly induces cell proliferation. (B) Claudin-18 siRNA-transfected MKN74 cells show a significantly higher invasion ratio compared to control or control siRNA-transfected cells. Typical images are presented at the top. Bar = 500 µm. Each value represents the mean ± SD of 3 independent experiments. **P<0.01 vs. control or control siRNA.

A GC cell line, MKN74, constitutively expressed claudin-18, and the mRNA level of claudin-18 was significantly reduced by siRNA against claudin-18 (Fig. 3A). Immunofluorescence staining with claudin-18 also decreased after transfection (Fig. 3B). In claudin-18 down-regulated MKN74, cell growth was significantly increased (Fig. 4A), and the invasion of the cells was significantly increased (Fig. 4B).

## Discussion

Altered expression of claudins at the submucosal invasive front might be related to the progression of GC. Recent studies suggest that even in slightly submucosal invasive GC without lymphatic and vascular invasion, ESD might be feasible as a curative operation [19]. However, it is still not clear which cases of slightly submucosal invasive GC can be followed without further surgery after ESD. In the present study, it was found that there was an inverse correlation between the claudin-18 level and Ki-67 positivity at the invasive front of submucosal invasive GC, and that claudin-18 regulated GC cell invasion and proliferation. Ki-67 immunohistochemistry has been substituted for mitotic counting in assessing tumor cell proliferation. Studies have demonstrated that Ki-67 is correlated with undifferentiated and metastatic cells in malignancies [20]. In this study, only EMR/ESD-resected GC in which the indication is limited to the differentiated type of GC was studied. Therefore, these data indicated that the Ki-67 level at the invasive front was related to metastatic cells in malignancies, and that loss of claudin-18 was one of the markers of GC aggressiveness and might be related to the development of metastasis, which necessitates additional surgery after ESD treatment.

Claudins are abnormally regulated in a variety of malignancies such as GC, hepatocellular carcinoma, biliary tract carcinoma, breast cancer, renal cell carcinoma, and mesothelioma [15], [21]–[25]. The change in claudins at junctions is associated with loss of tight adhesion and polarity in epithelia. Loss of polarity is associated with increased cellular proliferation and epithelial-to mesenchymal transition, while disruptions in tight junction complexes alter cell-cell interactions, which are related to invasion and metastasis [23], [26], [27]. In observational studies, decreases in claudin-3 and -4 have been reported in GC cells at the invasive front and were found to correlate with cancer progression and metastasis [14]. Down-regulation of claudins has also been reported in some other epithelial tumors, including loss of claudin-1 in breast cancer [28] and colonic cancer [29], as well as the loss of claudin-7 in breast cancer [30] and head and neck cancer [31]. Regarding the invasion of tumor cells, reduced expression of claudin-7 at the invasive front was found to be correlated with the depth of invasion and lymphatic involvement in squamous cell carcinoma of the esophagus [32]. Although claudin-3 and claudin-7 expressions were lower in the submucosal invasive front than in mucosal lesions in our previous study, claudin-3 and claudin-7 levels at the invasive front were not correlated with the Ki-67 LI [15]. On the other hand, claudin-18 at the invasive front was inversely correlated with the Ki-67 LI in the present study. These data may be related to the findings that claudin-3 and claudin-4 were not related to lymph node metastasis [33]. Furthermore, these observational studies cannot explain whether these changes directly affect tumor invasion and metastasis.

Claudin-18 is mainly expressed in stomach and lung epithelial cells. Claudin-18a2 is the form for stomach, and the isoforms are slightly different. In a previous report, gastric type claudin-18 was lost in GCs [17]. However, during intestinal metaplasia, the expression pattern of claudins already differs from that of normal gastric epithelia. The disappearance of claudin-18 was found to be associated with intestinal metaplasia in the stomach [17]. Therefore, the loss of claudin-18 expression in advanced GC [17] may not simply demonstrate the character of the cancer. Even in the process of GC progression, the expression level might change. To explore the function of these target molecules, we thought it was important to examine tumors at the same stage, and we evaluated the level of claudin-18, which might affect invasion and metastasis, in slightly submucosal invasive GC lesions. Furthermore, gastric mucosal claudin-18 may not work just at TJs, because the expressed localization on the cell surface was not just at TJs. Further investigations are needed to examine physiological and pathophysiological TJ protein expressions at the lateral membrane.

To examine whether the alterations in claudin-18 levels at the submucosal invasive front contributed to the proliferation and invasion of cancer cells, an siRNA approach was used to knockdown the protein in the MKN74 GC cell line, which is positive for claudin-18. Thus, the present study showed for first time the function of claudin-18 in GC using a genetic approach, and that loss of claudin-18 was involved in both proliferation and invasion of GC cells. Together with these data, loss of claudin-18 at the submucosal invasive front indicated the acquisition of more aggressive characteristics and might allow cell-cell dissociation during invasion. However, a limitation of this study was that only MKN74 could be examined, because it was the only differentiated gastric cancer cell line which expressed claudin-18 as far as we examined. Therefore, similar phenomena can not be reproduced with other gastric cancer cell lines.

Both up-regulation and down-regulation of claudins have been reported to be involved in the cancer process [34], [35]. For the analysis of the function of claudins in cancer cells, genetic approaches to claudins have also been reported in various cancer cells. Knockdown of claudin-1 by siRNA resulted in the inhibition of migration in colonic cancer cells [36]. The overexpression of claudin-6, claudin-7, or claudin-9 enhanced the invasive potential of a gastric adenocarcinoma cell line [37]. Such claudin-induced proliferation and invasion may seem paradoxical, because claudins are known to be involved in sealing epithelial junctions. These controversial data suggest that the functions of claudins are both subtype- and cell type-specific.

As a limitation of this study, it is still not clear that this decreased claudin-18 level at the submucosal invasive front is actually involved in cancer metastasis, because it is difficult to follow the natural history of early GCs. However, the data in this study indicated that a lower claudin-18 level at the invasive front was associated with greater proliferative tendency. It is necessary to prospectively examine whether this decrease in the claudin-18 level can be used as a metastatic and prognostic biomarker after endoscopic resection in a future study.

In summary, it has been shown for the first time that claudin-18 is heterogeneously expressed at the submucosal invasive front of early GC, and down-regulation of claudin-18 is associated with the proliferative and invasive potential of GC. These findings indicate that claudin-18 has a pivotal role in the progression of GC and may be a candidate biomarker of disease progression. A claudin-18 knockdown experiment confirmed that suppression of junctional claudin-18 promoted the proliferation and invasion of GC cells.
